# Trends in Austrian Resource Efficiency: An Exergy and Useful Work Analysis in Comparison to Material Use, CO_2_ Emissions, and Land Use

**DOI:** 10.1111/jiec.12474

**Published:** 2016-09-26

**Authors:** Nina Eisenmenger, Benjamin Warr, Andreas Magerl

**Affiliations:** Institute of Social Ecology, Alpen-Adria University, Schottenfeldgasse 29, 1070 Vienna, Austria

**Keywords:** Austria, exergy, energy analysis, resource efficiency, resource productivity, sustainable resource use

## Abstract

In the past few years, resource use and resource efficiency have been implemented in the European Union (EU) environmental policy programs as well as international sustainable development programs. In their programs, the EU focuses on four resource types that should be addressed: materials, energy (or carbon dioxide [CO_2_] emissions), water, and land. In this article, we first discuss different perspectives on energy use and present the results of a long-term exergy and useful work analysis of the Austrian economy for the period 1900–2012, using the methodology developed by Ayres and Warr. Second, we discuss Austrian resource efficiency by comparing the presented exergy and useful work data with material use, CO_2_ emissions, and land-use data taken from statistical sources. This comparison provides, for the first time, a long-term analysis of Austrian resource efficiency based on a broad understanding thereof and evaluates Austrian development in relation to EU and Austrian policy targets.

**Supplementary Information:**

The online version of this article (doi:10.1111/jiec.12474) contains supplementary material, which is available to authorized users.

## Introduction

Societies require natural resources to fuel socioeconomic processes from basic human and animal metabolism to industrial processes, transport activities, running electric devices, or building up infrastructure. The overall amount and structure of societal material and energy supply considered in relation to its use is described as industrial or social metabolism (Ayres and Ayres 2002; Ayres and Kneese 1969; Ayres and Simonis 1994; Fischer-Kowalski and Haberl 1993; Fischer-Kowalski and Hüttler 1998) and depends on the structures a society consists of. Over the course of history, we observe a changing material and energetic metabolism from 10 to 20 gigajoules (GJ) per capita for hunter-gatherer societies, over 40 to 70 GJ or 3 to 6 tonnes per capita for agrarian societies, and up to 150 to 400 GJ or 15 to 25 tonnes per capita for industrialized societies (Haberl 2006; Krausmann et al. 2008). The change from the agrarian to the industrial regime was mainly characterized by the introduction of fossil fuels, which made it possible to overcome the limitations of a bio-based society.

The twentieth century witnessed growing supplies of energy, but, at the same time, progress in energy technologies, which improved the efficiency of energy service delivery (Ayres et al. 2003). Very simply, it became possible to obtain more power from the same amount of energy at ever declining cost by technological innovation. This “technological progress^1^” stimulated a virtuous cycle of declining costs of energy leading to lower costs of energy-intensive products, subsequently stimulating increased consumption and revenues and by that growing economic activity. Economies react by increasing production and thus by benefiting from economies of scale. In parallel, investment in research and development (R&D) grows with efforts to develop new technologies to further reduce costs and improve the productivity. The consequence of these activities is increased economic growth and improved energy efficiency. In order to account for these “real” energy efficiencies, energy accounts are required that consider the technological transformation from primary energy resources to available exergy as the share that is potentially usable for humans and finally useful work as the actual energy service provided to societies. This is done by exergy accounts, which were published for the United States (Ayres et al. 2003), Sweden (Wall 1987), Japan (Williams et al. 2008), UK (Hammond and Stapleton 2001; Warr et al. 2008), China (Brockway et al. 2015), and the global level (Nakićenović et al. 1996); a comparative analysis was published for the United States, Japan, UK, and Austria (Warr et al. 2010), 15 European countries (Serrenho et al. 2014), and the United States and UK (Brockway et al. 2014). Building on the exergy accounts, researchers, first of all Ayres and Warr (2009), used exergy accounts to model economic growth, stating that “energy” is a driver of economic growth (Ayres et al. 2003; Ayres and Warr 2009; Voudouris et al. 2015; Warr et al. 2008, 2010; Williams et al. 2008; Hamilton 1983, 2003).

In recent years, the issue of resource efficiency prominently entered the policy arena. In 2011, the EU published a flagship initiative on a “resource efficient Europe” (EC 2012) followed by a roadmap guiding the implementation of the initiative (EC 2012). Similar policy documents were published by the Organization for Economic Cooperation and Development (OECD) (OECD 2004, 2012) and the United Nations Environment Program (UNEP 2012, 2012). Resource efficiency is broadening the perspective from energy toward including different types of resources (i.e., materials, energy, water, land, as well as carbon). With regard to resource use and efficiency, the European Union (EU) promotes a material use indicator as a headline indicator, that is, domestic material consumption (DMC) (EC 2012; Fischer-Kowalski et al. 2011), but recommends to complement it with a dashboard of indicators on energy, water, land, as well as carbon (EC 2012, 21).

This article first applies the exergy accounting method to Austria. The empirical database for Austria is updating the one used in Warr and colleagues (2010) until 2012^2^ and results in a data set of energy inputs^3^ for the period of 1900–2012; exergy inputs are allocated to categories of final use, and useful work supply is calculated by applying energy conversion efficiencies. In the second part, we are contrasting exergy and useful work with data on material use, carbon dioxide (CO_2_) emissions, and land use as well as the efficiencies derived thereof. By that, we apply the resource efficiency approach implemented by the European Commission (EC) to Austria and will discuss progress toward reaching policy targets.

## Methods and Data

### Exergy Analysis and Thermodynamics

Energy is contained within an energy carrier and conserved throughout all processes of transformation and use (a consequence of the first law of thermodynamics). When people talk about energy, however, they actually mean exergy^4^ (also understood as “available work” or “available energy”) because exergy is the useful part of energy that can be consumed and provides an “energy” service to societies. Different to energy, exergy is not conserved, but partially “used up” (Ayres and Warr 2009, xix ff). Before exergy can be used, it has to be converted to a usable form, which is termed useful work that is delivered to the point of final consumption. The different categories of useful work considered here are heat, light, mechanical power (used for moving vehicles), or electricity. The useful work is lower than the exergy entering the socioeconomic system because of transformation losses and thus a consequence of the second law of thermodynamics. The wasted fraction is dissipated into irreversible entropy generation. The fraction of the exergy that is lost depends on the efficiency of the transformation process and the technology used therein.

Exergy analysis has been applied to the understanding of ecosystem dynamics (Odum 1973; Schneider and Kay 1994), but also in the engineering of man-made systems and the design of efficient energy-using technologies. More recently, exergy has been used to assess changes in the supply, demand, and technology of regional and national economies. The majority of these studies focused on one single year, such as for the United States (Reistad 1975), Sweden (Wall 1987), Japan (Wall 1990), Canada (Rosen 1992), Italy (Wall et al. 1994), Turkey (Özdoĝan and Arikol 1995; Rosen and Dincer 1997), Norway (Ertesvåg and Mielnik 2000), the UK (Hammond and Stapleton 2001), Malaysia (Saidur et al. 2007), and a comparison across several countries all over the world (Ertesvåg 2001). More recently, studies have examined the historical evolution of resource exergy supply and utilization such as studies for China 1980–2002 (Chen and Chen 2007) and long-term studies of the twentieth century for the United States (Ayres et al. 2003), Japan (Williams et al. 2008), and the UK (Warr et al. 2008). The most recent work published data for the EU15 countries for the past 50 years (Serrenho et al. 2014) and for China (Brockway et al. 2015).

The transformation from natural resource exergy to useful work supply is a two-step process, which involves losses in the transformation as well as conversion processes. Transformation losses occur in the energy sector and refer to losses in the production of electricity from fossil fuels or of gasoline from crude oil. Conversion losses occur at the final use phase (i.e. households, etc.) and refer to the conversion of energy inputs to use equipment (such as furnaces, boilers, internal combustion engines, and electrical appliances, etc.) to energy services such as heat or light. Both losses depend on the efficiencies in the transformation or conversion processes and the technology applied therein. Considering these losses, we can calculate the useful work derived from the exergy input, differentiated according to the uses. We consider five categories of useful work: heat, light, mechanical drive (transport), and electricity. Electricity is considered “pure” useful work, which can perform either mechanical or chemical work with very high efficiency.

The database we compiled includes coal, crude oil and petroleum products, natural gas, and renewable resources (including biomass for feed of human workforce and draught animals) as exergy inputs. Data sources include historical data sets available for Austria for the years 1900–1960 (BMWA 1990; Bundesamt für Statistik 1925 and other years; Butschek et al. 1998; KAAW 1970; Krausmann et al. 2003) and for the years from the 1960s onward, data from the United Nations (UN) International Energy Agency (IEA) database were used (IEA 2011a, 2011b). Data and sources are presented in the Supporting Information available on the Journal’s website. Data availability and quality were problematic during the world war periods. For this reason, data for these years are not shown.

For fossil fuels, the primary exergy input is equivalent to the domestic energy supply. The conversion of other energy sources to exergy inputs follows the approach taken in previous studies by Ayres and Warr (Ayres et al. 2003; Warr et al. 2010). For biomass, the exergy inputs include fuel wood and biomass used to feed human workforce and draught animals. The biomass exergy inputs consumed are based on the estimation of daily energy intake and the efficiency of the food and feed processing systems developed by Wirsenius (2000). For more details on accounting methods, see the Supporting Information on the Web, as well as Ayres and Warr (2009) or Ayres and colleagues (2003).

The exergy inputs are then allocated to useful work categories representing the societal service derived from exergy inputs. Five useful work categories are differentiated (Ayres et al. 2003): (1) electricity, which we consider as pure work usable for all other purposes. (2) Prime movers, that is, all internal and external combustion engines such as steam turbines or jet engines. (3) Heat, which is further subdivided into three different temperature categories: high-temperature (HT) heat (>600°C) used for metal smelting or petroleum refining; medium-temperature (MT) heat (100 to 600°C) used to increase the solubility of solids in liquids; and low-temperature (LT) heat (<100°C) required for heating water or space, or for drying processes. We further subdivided LT in heat up to 20°C (for heating purposes), heat of around 40°C (mainly for drying purposes in the food and tobacco industry), and finally heat between 40 and 100°C (in other processes such as the pulp and paper industries). (4) Exergy use for light and (5) muscle work provided by draught animals and human workers.

Each type of final exergy consumption was allocated to the economic sectors differentiated in energy statistics. With regard to industrial heat requirements, further detail was needed that was not available from energy statistics. In that case, we used the same approach as in the study for the United States, which grouped half of all U.S. industrial process heat into high temperature and the other half to medium-temperature uses (Lovins 1977). We applied this approach to the coal, gas, and furnace oil used in the industrial sector; there was only one exemption (i.e., the iron and steel sector), where we assumed that all of the exergy going there is used for providing high-temperature heat. Flows to the residential and commercial sector were assumed to be primarily used to provide low-temperature (space) heat. This approach neglects other uses, such as space heating, mechanical drive, or electricity generation; however, we assume the potential bias in results to be minor.

Exergy losses incurred abroad in the production process of imported goods are not considered in this analysis; we followed a territorial perspective. In contrast, consumption-based accounts consider all resources that were globally used to satisfy domestic final consumption and thus include upstream resource uses of traded goods. Calculating consumption-based indicators is a methodologically difficult task; most studies use environmentally extended input-output models, which developed fast in recent years. Results for different resource types are available for single countries or country groups^5^ or for the whole world,^6^ which show that industrialized countries mostly increase their resource use if we change from a territorial- to a consumption-based perspective. This pattern also holds for Austria (see Schaffartzik et al. 2014a; Tukker et al. 2012). An application of a consumption-based approach was beyond the scope of this study, but is of high priority in future research. In the analysis presented here, we have to consider that changes in the Austrian efficiencies might be influenced by changes in the trade structures and outsourcing of exergy-intensive processes.

### Resource Use Measured as Material Use, Land Use, and Carbon Dioxide Emissions

Additional to exergy and useful work, we will use indicators from environmental accounts to provide a comprehensive picture of resource efficiency trends in Austria:

- *Material use*, measured as DMC, is the headline indicator of material flow accounts (Eurostat 2012, 2013; Fischer-Kowalski et al. 2011; Schaffartzik et al. 2014b). DMC is calculated as all materials extracted within Austria plus physical imports minus exports. All material flows are commonly aggregated to four material categories (biomass, fossil energy carriers, metal ores, and nonmetallic minerals) and measured in metric tonnes. DMC is also used as a proxy for resource use in general, such as in the European flagship initiative on a resource efficient Europe (EC 2012, 2011b).
- As a measure for *land use*, we use human appropriation of net primary production (HANPP) (Erb et al. 2009; Haberl et al. 2007, 2012), which is an aggregate indicator for land use and land-use intensification by measuring socioeconomic effects on ecological energy flows. HANPP measures changes in net primary production attributed to land conversion and biomass extraction through harvest.
- *CO*_*2*_ *emissions* measured as CO_2_ emissions from two sources, that is, from burning of fossil fuels and from manufacturing of cement, reported in metric tonnes (World Bank 2014).

## Austrian Exergy Inputs and Useful Work Outputs

### How Much Exergy Does the Austrian Economy Require to Fuel its Socioeconomic Processes? (Exergy Inputs)

**Figure 1:**
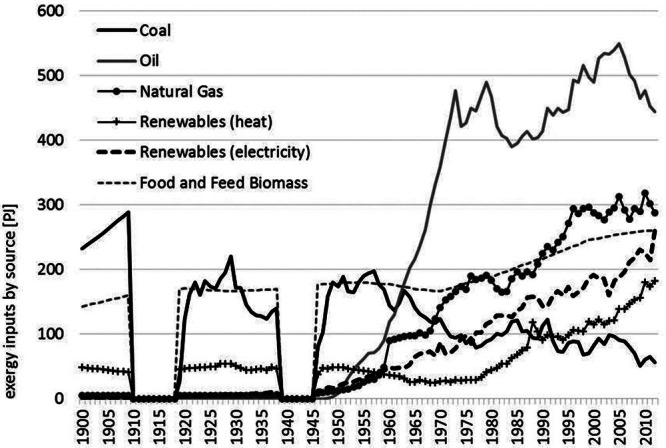
Primary exergy inputs (by source) in Austria in PJ, 1900 and 2012. *Note*: The world war periods are excluded for reasons of data quality. PJ = petajoules.

**Figure 2:**
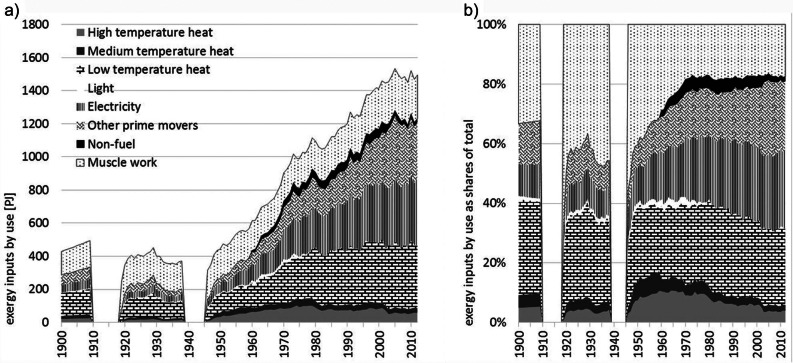
Exergy inputs in Austria by useful work type, 1900–2012: (a) total values in PJ and (b) shares of total. *Note*: The world war periods are excluded for reasons of data quality. PJ = petajoules.

Figure 1 shows the primary exergy inputs by source. At the start of the twentieth century, coal (60%; 230 petajoules [PJ]) and biomass (38%; 140 petajoules [PJ]) dominate the exergy supply mix. Over the century, their dominance declined down to 4% or 56 PJ for coal and 18% or 261 PJ for biomass in 2012. Currently, gas (19%; 288 PJ), oil (30%; 444 PJ), and commercial renewable energies (30%; 446 PJ) account for the majority of total supplies, which have increased from some 429 PJ in 1900 by a factor of 3.5 to 1,495 PJ in 2012. The contribution of renewable energies is dominated by hydroelectric power (190 PJ) providing electricity and biomass generating heat (see figure 2). At the end of the observed time period, we see that oil still remains to be the most important exergy source for Austria (30%).

Exergy inputs are used for different purposes, which we, according to Ayres and colleagues (2003), described along five types of useful work: electricity, prime movers, heat (HT, MT, and LT), light, and muscle work. Figure 2 shows the final exergy consumption by useful work type. The dominant trends over the century include the halving of muscle work, which accounted for 33% (143 PJ) of total consumption in 1900 and 17% (261 PJ) in 2012, and the dramatic increase in the use of electricity (10% [45 PJ] in 1900 to 27% [400 PJ] in 2012). The fraction of exergy consumed for heating has not changed a lot (38% [162 PJ] in 1900 and 31% [468 PJ] in 2012); however, this figure does not include the electricity subsequently used for heating, which we estimate as being roughly 30% of all electricity use.

Electricity developed to the most important energy use from the 1960s on. In Austria, most of the electricity is produced through hydropower and no nuclear power plant is operated. Useful work provided by electricity is a mixed category that can go to very different uses: One third goes to the industry sector and around 50% goes to commercial and public services and households. Six percent goes to the transport system (mainly railways) and 8% is used in the energy sector. Considering the useful work categories, electricity provides different services: lighting, cooling (refrigerators, air conditioning), industrial machinery, electronic devices, mobility (railways), etc. The use of electricity was constantly increasing up to 400 PJ in 2012, which is around 47 GJ per capita. In households and the governmental sector, electricity use is not very high, but characterized by highest growth rates (900% from 1960 to 2012). Any efficiency gains on the product level are overcompensated by a fast growing stock of electronic devices. Energy use for providing electricity is followed by the use categories, heat and transport. The latter even moved to second place in 2005, but from then on constantly dropped again down to the levels of 2003. The development for electricity and transport was characterized by pronounced growth since the 1950s, which led to an increase of useful work provided as electricity by a factor of 10 and as mechanical work for transport by a factor of 24.

### How Many Energy Services are Provided for Austrian Socioeconomic Processes? (Useful Work Outputs)

**Figure 3:**
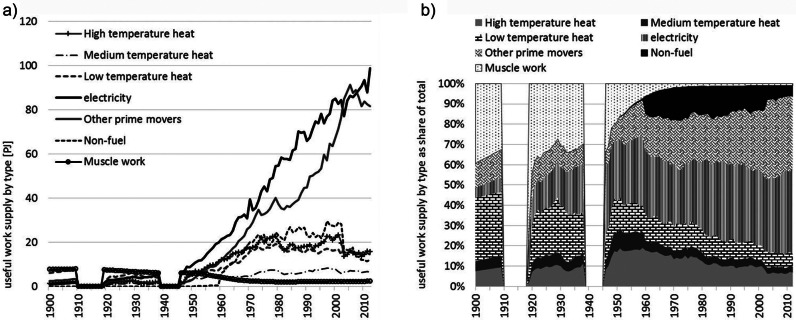
Useful work supply in Austria by type and share, 1900–2012: (a) total values in PJ and (b) shares of total. *Note*: The useful work supply in the category light was close to zero throughout the time period observed and thus excluded. The world war periods are excluded for reasons of data quality. PJ = petajoules.

Figure 3 presents the useful work supplied by type (figure 3a) and the share (figure 3b) of each type in the total. The declining importance of muscle work in providing power to the economy is now far more evident than when considered in terms of exergy inputs (see figure 2). The improvement in the efficiency with which fuel exergy is converted into useful work by machines has far exceeded the conversion efficiency within human bodies. A major trend is the electrification of the economy, with 45% (99 PJ) of all useful work supplied in 2012 by electrical devices. Electricity supplies increased rapidly by a factor of 10 since 1950. The useful work supplied for transport increased even faster (factor of 24), but the increase of the share in total was less marked as compared to electricity (14% [3 PJ] in 1900 to 37% [81 PJ] in 2012). Whereas the total useful work supply for heating has increased (from 8 to 37 PJ), the share in total has declined (40% to 17%).

**Figure 4:**
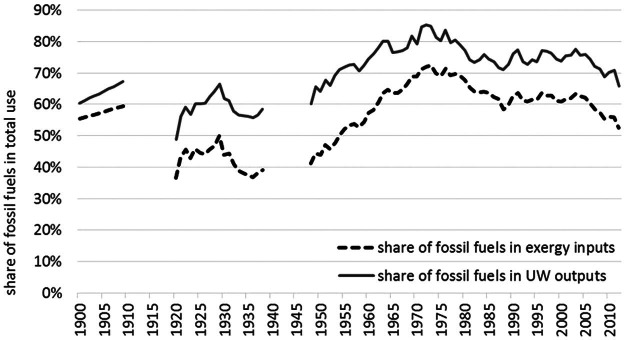
Fossil fuels as share of total exergy inputs and total useful work outputs in Austria. *Note*: The world war periods are excluded for reasons of data quality.

Despite the specific Austrian situation with the high importance of renewable energies, in particular, hydro power, Austria is still strongly depending on fossil fuels, which make up around 66% of useful work supplies or 53% of exergy inputs in 2012 (see figure 4). We also see that the importance of fossil fuels peaked in the early 1970s (around 70% of exergy inputs), declined until the late 1980s, and stayed at around 60% of exergy inputs until 2004. Only from 2005 onward, the share of fossil fuels declined again.

## Resource Efficiency of the Austrian Economy: How Does Austria Perform Under a Broader Perspective?

Resource efficiency is about providing a socioeconomic service (output) with least environmentally harmful inputs. We would like to stress two perspectives on resource efficiency: first, a technological efficiency, which is about relating physical outputs to physical inputs (i.e., minimizing primary inputs or wastes and emissions, but maximizing final use outputs). Second, we can assess economic efficiencies, mostly referred to as productivity, measuring the economic output produced in relation to a physical flow, which can either be resource inputs, but also physical outputs such as emissions.

### Technical Efficiencies

**Figure 5:**
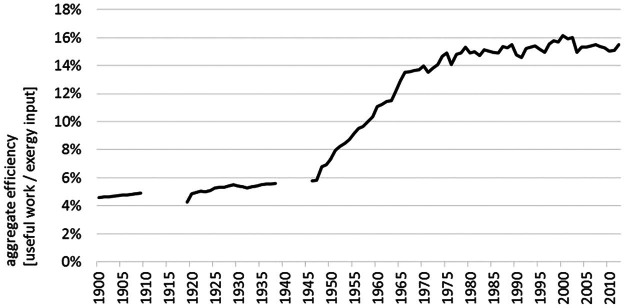
Aggregate efficiency of the conversion from exergy inputs to useful work in Austria, 1900–2012. *Note*: The world war periods are excluded for reasons of data quality.

We can estimate the aggregate energy conversion efficiency of the Austrian economy by dividing total useful work by total exergy inputs (see figure 5). Over the entire century, we observe a 3.3-fold improvement from 4.5 to 15%.^7^ The most rapid improvements, on average, 0.2% per annum, occurred in the postwar period between 1945 and 1980 as aging and damaged capital stock was replaced with the latest technologies, and the most efficient supplies (hydropower) were exploited. However, since 1980, there is evidence of a slowdown in the rate of improvement (0.06% per annum). The reasons for this slowdown are multiple and include an aging capital stock, the decrease in the total share of exergy supplies provided by hydropower, and an increase in the fraction of exergy used for transport.

The most marked efficiency improvements can be observed for high- and medium-temperature heat in industrial processes. The efficiency of electricity generation, distribution, and utilization has improved as well until 1980, but stagnated since then; there is even evidence of a decline because the fraction provided by hydroelectric generation has declined relative to fossil fuels. The exergy efficiency of transportation has doubled until 1970. Since then, no significant improve can be observed. This change in efficiency coincides with the point in time when gasoline engines operate at higher compression ratios. The exergy efficiency of low-temperature heat processes follows a different development path of much slower increases.

**Figure 6:**
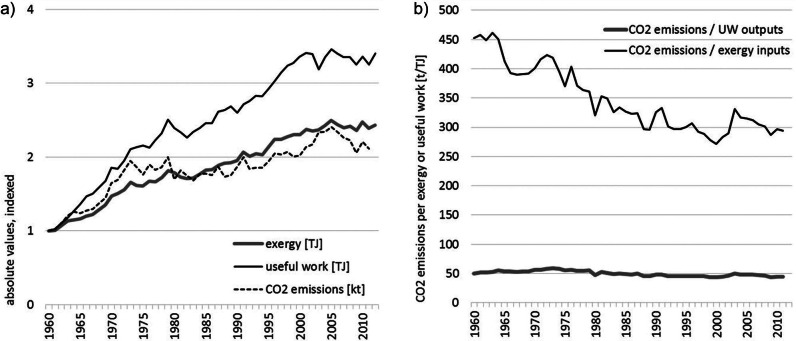
Exergy and useful work in relation to CO_2_ emissions: (a) absolute values, indexed, and (b) CO_2_ emissions in relation to exergy inputs and useful work outputs, Austria 1960-2012. *Note*: Exergy and useful work: own calculations; CO_2_ emissions (World Bank 2014). CO_2_ = carbon dioxide.

In the process of burning of fossil fuels, CO_2_ emissions are produced, which is why both develop at similar trends (see figure 6a). However, differences can occur because of changes in the fuel mix (i.e., the energy carriers used), which induce different CO_2_ emissions. In 1960, coal was the major energy carrier (1960: 23% of exergy inputs derived from coal, 19% from oil, and 15% from natural gas), but its share decreased constantly in the following 15 years. In 2012, coal only made up 4% of exergy inputs, whereas oil (30%) and gas (19%) where then the main energy carriers used. Nevertheless, the CO_2_ emissions caused by each unit of exergy inputs used stayed rather constant over the past 50 years. Technology gains in the conversion of exergy inputs to useful work outputs lead to a decoupling of useful work outputs from exergy inputs and thus from CO_2_ emissions (see figure 5 and figure 6b). Whereas CO_2_ emissions per unit of exergy inputs only declined slightly (12% between 1960 and 2012), CO_2_ emissions per unit of useful work output decreased by 35% during the same time period, indicating a decline of the environmental burden induced by the energy service required by the Austrian society.

### Austrian Resource Productivity: A Dashboard of Indicators

Throughout the entire century, the economic productivity of exergy inputs has constantly increased, as indicated by the gross domestic product (GDP)/exergy ratio, which increased at 1.1% per annum, comparable to that of the United States at 1.2% per annum (Ayres et al. 2003). Each unit of exergy consumed in 2012 is twice as productive as it was in 1900.

Despite the slowdown in technical efficiency improvements since the 1980s, figure 7 reveals that only since 1980 the Austrian economy improves its economic efficiency of useful work (as indicated by the increasing GDP/useful work ratio), whereas between 1940 and 1980 the useful work efficiency declined. In 1940, 1.1 terajoules (TJ) of useful work equates to 1 million 1990 Geary–Khamis dollars (GK$), by 1980 the Austrian economy used 1.3 TJ useful work for producing the same 1 million 1990 GK$. From 1980 onward, the trend changed toward an improvement, which reduced the ratio by 0.5% per annum to again 1.1 TJ per 1 million 1990 GK$ in 2012. The rate of decline is slightly slower than the observed rate for the UK (1.4% per annum) (Warr et al. 2008). Given the slowdown in the improvements in technical efficiency, we hypothesize that progress in the economic efficiency of useful work is the result of a shift in the structure of the Austrian economy, away from low value added and highly energy-intensive industries toward an economy dominated by service sectors. A change in trade structure (i.e., using fewer energy-intensive processes domestically) might even add to this development, however, needs to be tested with empirical data on the consumption-based perspective (see *Methods and Data*). The timing of this “structural shift,” coinciding with the oil crises and energy concerns of the early 1970s and 1980s, suggests that the two events are not unrelated. It is likely that the energy price spike has had a persistent long-term effect on the structure of useful work demand and its relation to economic activity.

**Figure 7:**
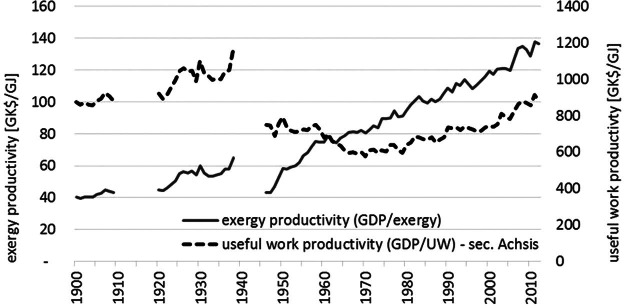
Exergy and useful work productivities in Austria, 1900–2012. *Note*: The world war periods are excluded for reasons of data quality.

**Figure 8:**
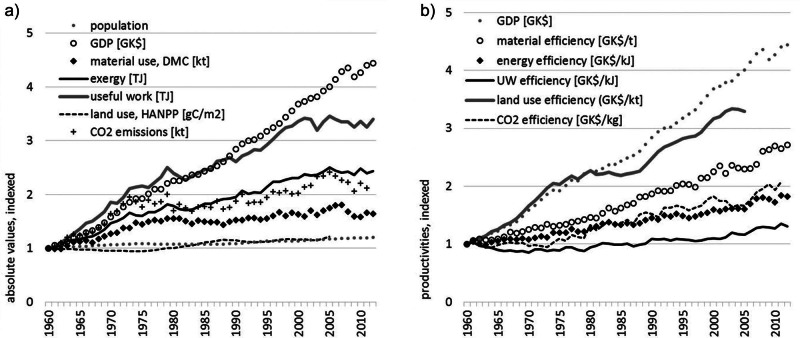
Development of resource use and resource efficiency in Austria, 1960–2012: (a) absolute values, indexed, and (b) productivities. *Data and sources*: population (1,000 persons) from FAOSTAT (FAO 2012); GDP (million Geary-Khamis dollars; World Economics [2012]); exergy and useful work: own calculations; domestic material consumption (DMC) (million metric tonnes/yr) from Statistics Austria (2012) and material efficiency = GDP/DMC (GK$/t); human appropriation of net primary production (HANPP) (g C/m^2^/yr) from Gingrich and colleagues (2015) and Krausmann and colleagues (2012) and land-use efficiency measured as GDP/HANPP (GK$/kt/m^2^); HANPP data are only available until 2005; CO_2_ emissions (kt) (World Bank 2014) and CO_2_ efficiency (GK$/kg); no CO_2_ emission data available for 2012. CO_2_ = carbon dioxide; g C/m^2^/yr = grams of carbon per square meter per year; GDP = gross domestic product; GK$/t = Geary–Khamis dollars per tonne; kg = kilogram; kt = kilotonnes; yr = year.

Energy addresses only a specific aspect of resource use. Broadening the perspective toward other resources and environmental issues helps in getting a better picture of problem areas and sustainable development in general. In the following, we will contrast energy-use data with other indicators on resource use, that is, material use (with the indicator, DMC; for concepts and methods, see Eurostat [2013]; Fischer-Kowalski et al. [2011]), land-use intensity (represented by the indicator, HANPP; for concepts and methods, see Erb et al. [2009]; Haberl et al. [2007, 2012]), and CO_2_ emissions as a representative for the physical outputs of societies. The selection of indicators follows the proposal of the EU in their description of a “dashboard of indicators” to monitor a development toward a resource-efficient Europe (EC 2012).

Between 1960 and 2012, GDP was constantly growing (factor of 4.4) only with a short recession phase during the economic crises in 2008 and 2009. All resource use indicators are growing as well, indicating that Austria is using more resources of all kinds. Highest growth rates are observed for useful work outputs (factor of 3.4). Increases in the use of exergy inputs or the emission of CO_2_ develop at a similar pace (factor of 2.4 and 2.1, respectively) and lowest growth rates are observed for material use (DMC; factor of 1.6). Finally, the measure for land-use intensity (i.e., HANPP) shows nearly no growth (only +22%), indicating that the Austrian economy is not increasing its pressure on land significantly.

For all resource use indicators, a decoupling from economic growth is observed, that is, resource use grows at slower rates as compared to GDP. However, none of the resource use indicators follow a declining trend, which would indicate an absolute decrease of the pressure on the domestic environment. Highest productivity gains are observed for the GDP to land-use ratio (GDP/HANPP; factor of 3.3) and for material productivity (GDP/DMC; factor of 2.7). Productivities in relation to CO_2_ emissions and exergy develop at a similar pace (factor of 2.1 and 1.8, respectively) and smallest productivity gains are observed to the GDP/useful work output ratio (factor of 1.3).

Let us now evaluate the Austrian trends in relation to national and European policy programs and targets defined therein. With regard to energy and climate change, the EU has published the Energy Strategy (EC 2012) with three goals defined: (1) reduce greenhouse gas emissions by 20%; (2) increase the share of renewable energy to 20%; and (3) improve the energy efficiency by 20%. All targets are referring to the base year 1990 and should be reached by 2020. Concerning the first target, Austria has not yet reduced CO_2_ emissions, but increased CO_2_ emissions by 13% between 1990 and 2012. With regard to target (2), the exergy analysis presented above showed that the share of renewable energy is relatively high in Austria attributed to the large share of hydropower: In 2012, 30% of the exergy inputs were derived from renewable sources, which exceeds the requirements of the EU program. Austrian energy efficiency as addressed by target (3) increased by 25% between 1990 and 2020. However, efficiency gains are mainly based on GDP growth, but no absolute reduction of energy use (neither in exergy inputs, nor in useful work outputs) could be achieved.

With regard to the material use, the EU is currently discussing possible targets, but did not yet agree on a particular one. In contrast, on the national level, the Austrian Federal Ministry of Agriculture, Forestry, Environment and Water Management published a resource efficiency action plan in 2012 (BM LFUW 2012) with the following targets: Material productivity should increase by 50% between 2008 and 2020 at a reduction of DMC by 20%. Data on material use reveals that Austria so far failed to reach the addressed targets: Between 2008 and 2020, DMC decreased by only 2%, resulting in an increase of material efficiency by only 4%.

## Conclusions

Energy is a prerequisite for industrial production and modern lifestyle patterns. During the twentieth century, changes in energy-use patterns accelerated and led to a significant increase of the amount of energy we use, but also to a change in the structure and composition of the socioeconomic energy basis. But an energy analysis has to be more specific: Only part of the exergy input can actually be used because of losses in the energy transformation process and because of technological limitations and inefficiencies in the use phase. The fraction that is left after transformation and losses and remains for satisfying our final demands is the “useful work” fraction. A ratio of the exergy input to the useful work portrays the economy-wide efficiency of transformation and use. At the same time, the ratio shows the amount of exergy input that is wasted during the socioeconomic processing. The results for Austria show that only 15% of the actual available natural resource exergy input we are acquiring from nature were delivered to the economy as useful work. Over the twentieth century, the increase of the aggregated efficiency was remarkable; from 5% in 1900 and arrived at the above-mentioned 15% in 2012. However, the percentage is still low, indicating that we are losing 85% of the natural resource exergy input. The question is how much technical progress can be expected to further increase efficiency and where are the limits to this?

The analysis of the exergy inputs grouped along the final use categories revealed that electricity is the major and most strongly growing use category. We must note, however, that this electricity is used to provide energy services of heating, lighting, etc., that is, contributing to the other use categories as well. The major fraction of electricity goes to households and commercial and public services; the remainder (30%) goes to industrial production. Highest growth is given for the residential and commercial sectors, a reflection of the increasing electrification of services and households, improved standards of living, and dominance of the service sector in the economy.

In Austria, most of the electricity is produced by hydro power plants. Hydro power is considered as a renewable energy source that has a very high efficiency in converting exergy inputs to useful work. More than 50% of all useful work is provided as electricity. The second highest fraction of useful work consumption in 2012 is for transport uses. Both sources of demand are accelerating; however, historical sources of exergy meeting this demand are potentially constrained. It is widely accepted that oil will become scarce in the future, indicating that an alternative exergy source must be sought to meet transport requirements. Also, Austria cannot rely on increasing sources of hydro power for electricity production given that the majority of suitable sites have already been exploited. This begs the question; what are suitable alternative sources of exergy? Moreover, how can the socioeconomic metabolism of Austria be restructured, and the dominant technologies used replaced to ensure adequate delivery of energy services in the future?

The exergy productivity of GDP was constantly increasing over the twentieth century, a reflection of the technological progress that enabled more useful work to be delivered per unit of exergy consumed. In contrast, the useful work productivity of GDP displays three phases: slightly increasing productivity until the end of World War II (WWII), which was followed by a phase of rapid decline until the end of the 1960s followed by stabilization, and finally a phase of increasing productivity from the early 1970s onward. This indicates that the Austrian economy became increasingly less efficient during the first 70 years of the twentieth century, in particular, in the post-WWII period when the economy was being rebuilt. It also shows that efficiency gains at the end of the century were not large. The timing of the turnaround in this trend, occurring in the early 1970s (as it does for other countries studied, the United States, UK, and Japan), indicates that the oil crises and rapid increases in energy costs stimulated an increase in the efficiency of useful work consumption that has been permanent. It is very likely that pressures to improve resource use efficiency stemming from the need to reduce greenhouse gas emissions associated with energy use have contributed to making this declining trend in useful work intensity a permanent feature of the economy.

A broader analysis of resource efficiency based on indicators on material use, land use, and CO_2_ emissions revealed that Austria’s efficiency increases in energy use are even outpaced by other resource efficiency measures: Land-use intensity did not change much in recent years, leading to a high land-use efficiency. Material productivity increased moderately attributed to a stagnating material use, in particular, since the early 1970s. However, efficiency gains are driven by economic growth; resource use did slow down, but not decrease, resulting in a relative decoupling rather than absolute decoupling of resource use from GDP growth. The trends in resource use and resource efficiency allow Austria to meet the requirements of the European Energy Strategy, but fail in meeting the Austrian target on material productivity as defined in the Austria Resource Efficiency Action Plan. Effects of a changing trade structure and a possible shift of resource-intensive processes to other countries on Austrian resource efficiency are not considered here, but need to be analyzed in the future.

## Supplementary Information

**Supporting Information S1**: This supporting information provides details on data and data sources used in the exergy account. This includes in particular: 1) data tables on exergy inputs by energy carrier and by use, as well as useful work supply by type, and 2) data sources with a detailed description of historical data sources used and a comparison of the data from different sources.

## Footnotes

1 Clearly, improvements in technology are the end result of a long process of R&D, invention, innovation, and widespread application necessarily stemming from improvements in the knowledge base of society. We acknowledge that enhanced knowledge capacity is an essential component of the technology drive. However, we consider that it is not possible to derive a measure of knowledge capital.

2 Slight deviations from the data published in 2010 occur because of minor changes in the primary data as well as revisions of the accounting and estimation methods.

3 For a discussion of exergy, see the subsequent section.

4 By definition, the exergy of a system or a resource is the maximum amount of useful work that can be obtained from this system or resource when it is brought to equilibrium uniquely with its surroundings through reversible processes. For a detailed discussion of the methods of exergy estimation, see Szargut and colleagues (1988). Thus, exergy “is not a simple thermodynamic property, but rather a co-property of a system and the reference environment” (Dincer and Cengel 2001, 129). Different to that, entropy is a property of a system. “When a system is isolated, energy increase will be zero. In this case the entropy of the system will continue to increase due to irreversible processes and reach the maximum possible value, which is the state of thermodynamic equilibrium” (Dincer and Cengel 2001, 122). Low entropy is considered “useful” energy, whereas high entropy is a rather unusable form of energy. For more details on definitions of exergy, energy, and entropy within the laws of thermodynamics, see Dincer and Cengel (2001).

5 E.g. (Muñoz et al. 2009; Schaffartzik et al. 2014a; Schoer et al. 2012; Wood and Dey 2009; Bruckner et al. 2012; Wiebe et al. 2012; Kastner et al. 2014).

6 E.g. (Wiedmann et al. 2015; Tukker et al. 2012; Peters et al. 2011; Hertwich and Peters 2009; Hoekstra and Chapagain 2006; Hoekstra and Mekonnen 2012; UNEP 2012).

7 Serrenho and colleagues (2014) result in a similar, but higher efficiency of 21% in 2010.
