# Supplementary material for: Trends in Austrian Resource Efficiency: An Exergy and Useful Work Analysis in Comparison to Material Use, CO2 Emissions, and Land Use
Source: J Ind Ecol. 2016 Sep 26;21(5):1250–61. doi: 10.1111/jiec.12474 (PMC5763337; doi:10.1111/jiec.12474)
Supplement: Supplementary file 1 — Supporting Information S1: This supporting information provides details on data and data sources used in the exergy account. This includes in particular: 1) data tables on exergy inputs by energy carrier and by use, as well as useful work supply by type, and 2) data sources with a detailed description of historical data sources used and a comparison of the data from different sources. [file 44498_2017_2105017_MOESM1_ESM.pdf]

SUPPORTING INFORMATION FOR:

Eisenmenger, N., B. Warr, and A. Magerl. 2016. Trends in Austrian resource efficiency. An exergy and useful work analysis in comparison to material use, $CO_2$ emissions, and land use. *Journal of Industrial Ecology.*

**Summary**

This supporting information provides details on data and data sources used in the exergy account. This includes in particular: 1) data tables on exergy inputs by energy carrier and by use, as well as useful work supply by type; and 2) data sources with a detailed description of historical data sources used and a comparison of the data from different sources.

# Data

| Table S1: exergy inputs by energy carrier, 1900-2012 [units: PJ] | | | | | | |
|---|---|---|---|---|---|---|
| year | total | Coal | Oil | Gas | Renewables | Biomass |
| 1900 | 429 | 232 | 0 | 5 | 48 | 143 |
| 1901 | 436 | 238 | 0 | 5 | 48 | 145 |
| 1902 | 443 | 245 | 0 | 5 | 47 | 146 |
| 1903 | 449 | 250 | 0 | 5 | 46 | 148 |
| 1904 | 457 | 256 | 0 | 5 | 45 | 150 |
| 1905 | 464 | 262 | 0 | 5 | 44 | 152 |
| 1906 | 472 | 269 | 0 | 5 | 43 | 154 |
| 1907 | 479 | 276 | 0 | 5 | 42 | 156 |
| 1908 | 487 | 282 | 0 | 5 | 42 | 158 |
| 1909 | 495 | 289 | 0 | 5 | 41 | 160 |
| 1910 | | | | | | |
| 1911 | | | | | | |
| 1912 | | | | | | |
| 1913 | | | | | | |
| 1914 | | | | | | |
| 1915 | | | | | | |
| 1916 | | | | | | |
| 1917 | | | | | | |
| 1918 | | | | | | |
| 1919 | 251 | 27 | 0 | 5 | 49 | 170 |
| 1920 | 348 | 122 | 0 | 5 | 50 | 170 |
| 1921 | 388 | 162 | 0 | 5 | 51 | 170 |
| 1922 | 407 | 180 | 0 | 5 | 52 | 170 |
| 1923 | 388 | 161 | 0 | 5 | 53 | 169 |
| 1924 | 410 | 182 | 0 | 5 | 54 | 169 |
| 1925 | 402 | 174 | 0 | 5 | 55 | 168 |
| 1926 | 399 | 171 | 0 | 5 | 56 | 168 |
| 1927 | 418 | 185 | 0 | 5 | 60 | 167 |
| 1928 | 427 | 195 | 0 | 5 | 61 | 167 |
| 1929 | 453 | 221 | 0 | 5 | 61 | 166 |
| 1930 | 401 | 171 | 0 | 5 | 58 | 166 |
| 1931 | 399 | 171 | 0 | 5 | 55 | 167 |
| 1932 | 369 | 146 | 0 | 5 | 51 | 167 |
| 1933 | 358 | 134 | 0 | 5 | 51 | 167 |
| 1934 | 356 | 129 | 0 | 6 | 53 | 168 |
| 1935 | 356 | 128 | 0 | 5 | 54 | 168 |
| 1936 | 350 | 123 | 0 | 5 | 53 | 169 |
| 1937 | 367 | 135 | 0 | 5 | 58 | 169 |
| 1938 | 370 | 139 | 0 | 6 | 56 | 169 |
| 1939 | | | | | | |
| 1940 | | | | | | |
| 1941 | | | | | | |
| 1942 | | | | | | |
| 1943 | | | | | | |
| 1944 | | | | | | |
| 1945 | | | | | | |
| 1946 | 316 | 82 | 0 | 9 | 50 | 176 |
| 1947 | 349 | 102 | 0 | 10 | 60 | 177 |
| 1948 | 411 | 158 | 1 | 11 | 64 | 177 |
| 1949 | 437 | 180 | 3 | 11 | 65 | 178 |
| 1950 | 441 | 173 | 9 | 12 | 69 | 179 |
| 1951 | 473 | 189 | 20 | 14 | 71 | 179 |
| 1952 | 465 | 166 | 32 | 15 | 72 | 180 |
| 1953 | 479 | 165 | 45 | 19 | 71 | 179 |
| 1954 | 508 | 180 | 54 | 21 | 74 | 179 |
| 1955 | 532 | 190 | 61 | 26 | 75 | 180 |
| 1956 | 549 | 194 | 70 | 29 | 76 | 179 |
| 1957 | 556 | 197 | 72 | 31 | 78 | 178 |
| 1958 | 550 | 176 | 77 | 36 | 82 | 178 |
| 1959 | 567 | 169 | 90 | 49 | 81 | 177 |

**Table S1 (continued): exergy inputs by energy carrier, 1900-2012 [units: PJ]**

| year | total | Coal | Oil | Gas | Renewables | Biomass |
|---|---|---|---|---|---|---|
| 1960 | 614 | 144 | 119 | 90 | 85 | 177 |
| 1961 | 618 | 137 | 130 | 92 | 83 | 176 |
| 1962 | 657 | 149 | 156 | 95 | 83 | 174 |
| 1963 | 695 | 167 | 179 | 97 | 79 | 173 |
| 1964 | 707 | 157 | 202 | 98 | 78 | 171 |
| 1965 | 714 | 141 | 216 | 98 | 90 | 170 |
| 1966 | 735 | 131 | 235 | 102 | 98 | 169 |
| 1967 | 752 | 126 | 261 | 98 | 98 | 168 |
| 1968 | 789 | 120 | 300 | 105 | 98 | 167 |
| 1969 | 829 | 117 | 331 | 124 | 92 | 166 |
| 1970 | 903 | 123 | 358 | 140 | 114 | 167 |
| 1971 | 926 | 107 | 400 | 153 | 97 | 169 |
| 1972 | 958 | 96 | 435 | 159 | 97 | 171 |
| 1973 | 1 019 | 94 | 477 | 166 | 108 | 173 |
| 1974 | 991 | 99 | 421 | 174 | 121 | 176 |
| 1975 | 986 | 86 | 427 | 170 | 126 | 178 |
| 1976 | 1 028 | 94 | 450 | 190 | 114 | 181 |
| 1977 | 1 023 | 79 | 446 | 184 | 132 | 183 |
| 1978 | 1 060 | 82 | 469 | 187 | 136 | 185 |
| 1979 | 1 113 | 88 | 490 | 190 | 157 | 188 |
| 1980 | 1 096 | 92 | 468 | 183 | 164 | 189 |
| 1981 | 1 060 | 101 | 421 | 171 | 177 | 192 |
| 1982 | 1 048 | 98 | 407 | 165 | 183 | 194 |
| 1983 | 1 053 | 104 | 403 | 166 | 183 | 197 |
| 1984 | 1 084 | 120 | 390 | 185 | 189 | 200 |
| 1985 | 1 121 | 121 | 395 | 196 | 205 | 203 |
| 1986 | 1 122 | 104 | 406 | 190 | 216 | 206 |
| 1987 | 1 160 | 105 | 414 | 197 | 235 | 209 |
| 1988 | 1 177 | 94 | 402 | 192 | 276 | 212 |
| 1989 | 1 181 | 93 | 404 | 208 | 261 | 215 |
| 1990 | 1 200 | 112 | 413 | 225 | 233 | 217 |
| 1991 | 1 269 | 122 | 449 | 236 | 242 | 220 |
| 1992 | 1 234 | 89 | 439 | 230 | 254 | 223 |
| 1993 | 1 255 | 74 | 450 | 242 | 263 | 226 |
| 1994 | 1 246 | 72 | 443 | 251 | 251 | 229 |
| 1995 | 1 310 | 87 | 448 | 272 | 272 | 232 |
| 1996 | 1 376 | 88 | 493 | 294 | 264 | 235 |
| 1997 | 1 374 | 88 | 490 | 287 | 271 | 239 |
| 1998 | 1 396 | 68 | 516 | 294 | 276 | 242 |
| 1999 | 1 416 | 70 | 497 | 297 | 306 | 245 |
| 2000 | 1 414 | 82 | 490 | 288 | 307 | 247 |
| 2001 | 1 459 | 93 | 526 | 283 | 308 | 248 |
| 2002 | 1 446 | 85 | 534 | 277 | 300 | 250 |
| 2003 | 1 452 | 98 | 533 | 288 | 280 | 251 |
| 2004 | 1 488 | 96 | 540 | 295 | 304 | 253 |
| 2005 | 1 535 | 91 | 549 | 313 | 327 | 254 |
| 2006 | 1 499 | 89 | 528 | 292 | 335 | 256 |
| 2007 | 1 473 | 80 | 502 | 278 | 357 | 257 |
| 2008 | 1 485 | 71 | 492 | 294 | 371 | 258 |
| 2009 | 1 451 | 51 | 465 | 289 | 387 | 259 |
| 2010 | 1 522 | 61 | 476 | 319 | 407 | 259 |
| 2011 | 1 468 | 65 | 453 | 302 | 387 | 260 |
| 2012 | 1 495 | 56 | 444 | 288 | 446 | 261 |

| Table S2: exergy inputs by use, 1900-2012 [units: PJ] | | | | | | | | | |
|---|---|---|---|---|---|---|---|---|---|
| year | total | high temp. heat | medium temp. heat | low temp. heat | light | electricity | other prime movers | non-fuel | muscle work |
| 1900 | 429 | 21 | 18 | 138 | 5 | 45 | 59 | 0 | 143 |
| 1901 | 436 | 22 | 19 | 139 | 5 | 46 | 61 | 0 | 145 |
| 1902 | 443 | 22 | 19 | 141 | 5 | 47 | 62 | 0 | 146 |
| 1903 | 449 | 23 | 19 | 142 | 5 | 48 | 64 | 0 | 148 |
| 1904 | 457 | 23 | 20 | 144 | 5 | 49 | 65 | 0 | 150 |
| 1905 | 464 | 24 | 20 | 145 | 5 | 50 | 67 | 0 | 152 |
| 1906 | 472 | 24 | 21 | 147 | 5 | 52 | 68 | 0 | 154 |
| 1907 | 479 | 25 | 22 | 149 | 5 | 53 | 70 | 0 | 156 |
| 1908 | 487 | 26 | 22 | 150 | 5 | 54 | 72 | 0 | 158 |
| 1909 | 495 | 26 | 23 | 152 | 5 | 55 | 74 | 0 | 160 |
| 1910 | | | | | | | | | |
| 1911 | | | | | | | | | |
| 1912 | | | | | | | | | |
| 1913 | | | | | | | | | |
| 1914 | | | | | | | | | |
| 1915 | | | | | | | | | |
| 1916 | | | | | | | | | |
| 1917 | | | | | | | | | |
| 1918 | | | | | | | | | |
| 1919 | 251 | 2 | 2 | 56 | 5 | 9 | 7 | 0 | 170 |
| 1920 | 348 | 11 | 10 | 93 | 5 | 27 | 31 | 0 | 170 |
| 1921 | 388 | 15 | 13 | 109 | 5 | 35 | 41 | 0 | 170 |
| 1922 | 407 | 16 | 14 | 117 | 5 | 39 | 46 | 0 | 170 |
| 1923 | 388 | 15 | 13 | 110 | 5 | 35 | 41 | 0 | 169 |
| 1924 | 410 | 17 | 14 | 119 | 5 | 40 | 46 | 0 | 169 |
| 1925 | 402 | 18 | 15 | 114 | 5 | 38 | 44 | 0 | 168 |
| 1926 | 399 | 17 | 14 | 114 | 5 | 37 | 45 | 0 | 168 |
| 1927 | 418 | 18 | 15 | 124 | 5 | 42 | 47 | 0 | 167 |
| 1928 | 427 | 20 | 17 | 130 | 5 | 44 | 46 | 0 | 167 |
| 1929 | 453 | 21 | 18 | 145 | 5 | 48 | 51 | 0 | 166 |
| 1930 | 401 | 17 | 15 | 114 | 5 | 43 | 40 | 0 | 166 |
| 1931 | 399 | 14 | 13 | 122 | 5 | 38 | 40 | 0 | 167 |
| 1932 | 369 | 11 | 10 | 107 | 5 | 37 | 31 | 0 | 167 |
| 1933 | 358 | 11 | 10 | 100 | 5 | 35 | 30 | 0 | 167 |
| 1934 | 356 | 11 | 10 | 97 | 5 | 34 | 30 | 0 | 168 |
| 1935 | 356 | 13 | 11 | 98 | 5 | 31 | 29 | 0 | 168 |
| 1936 | 350 | 13 | 12 | 91 | 5 | 33 | 27 | 0 | 169 |
| 1937 | 367 | 14 | 12 | 103 | 5 | 34 | 30 | 0 | 169 |
| 1938 | 370 | 16 | 14 | 97 | 6 | 37 | 31 | 0 | 169 |
| 1939 | | | | | | | | | |
| 1940 | | | | | | | | | |
| 1941 | | | | | | | | | |
| 1942 | | | | | | | | | |
| 1943 | | | | | | | | | |
| 1944 | | | | | | | | | |
| 1945 | | | | | | | | | |
| 1946 | 316 | 9 | 8 | 67 | 7 | 30 | 18 | 0 | 176 |
| 1947 | 349 | 13 | 11 | 80 | 7 | 37 | 24 | 0 | 177 |
| 1948 | 411 | 25 | 20 | 100 | 8 | 50 | 32 | 0 | 177 |
| 1949 | 437 | 32 | 25 | 108 | 7 | 54 | 32 | 0 | 178 |
| 1950 | 441 | 33 | 26 | 110 | 7 | 51 | 35 | 0 | 179 |
| 1951 | 473 | 41 | 32 | 113 | 7 | 58 | 41 | 0 | 179 |
| 1952 | 465 | 39 | 29 | 109 | 7 | 57 | 44 | 0 | 180 |
| 1953 | 479 | 41 | 30 | 112 | 8 | 62 | 47 | 0 | 179 |
| 1954 | 508 | 44 | 32 | 120 | 8 | 71 | 52 | 1 | 179 |
| 1955 | 532 | 49 | 35 | 126 | 9 | 78 | 56 | 1 | 180 |
| 1956 | 549 | 52 | 38 | 128 | 10 | 82 | 60 | 1 | 179 |
| 1957 | 556 | 54 | 38 | 128 | 10 | 88 | 58 | 1 | 178 |
| 1958 | 550 | 54 | 37 | 121 | 11 | 90 | 60 | 1 | 178 |
| 1959 | 567 | 60 | 38 | 121 | 11 | 93 | 65 | 1 | 177 |

| Table S2 (continued): exergy inputs by use, 1900-2012 [units: PJ] | | | | | | | | | |
|---|---|---|---|---|---|---|---|---|---|
| year | total | high temp. heat | medium temp. heat | low temp. heat | light | electricity | other prime movers | non-fuel | muscle work |
| 1960 | 614 | 64 | 26 | 150 | 14 | 97 | 73 | 12 | 177 |
| 1961 | 618 | 64 | 25 | 142 | 15 | 106 | 77 | 14 | 176 |
| 1962 | 657 | 66 | 27 | 159 | 16 | 113 | 84 | 18 | 174 |
| 1963 | 695 | 70 | 30 | 176 | 17 | 120 | 91 | 20 | 173 |
| 1964 | 707 | 76 | 33 | 167 | 17 | 126 | 96 | 21 | 171 |
| 1965 | 714 | 76 | 33 | 168 | 18 | 126 | 100 | 23 | 170 |
| 1966 | 735 | 78 | 34 | 167 | 17 | 134 | 108 | 28 | 169 |
| 1967 | 752 | 76 | 32 | 181 | 17 | 135 | 113 | 29 | 168 |
| 1968 | 789 | 81 | 32 | 201 | 17 | 141 | 118 | 31 | 167 |
| 1969 | 829 | 86 | 32 | 211 | 19 | 146 | 131 | 36 | 166 |
| 1970 | 903 | 82 | 31 | 247 | 21 | 183 | 132 | 40 | 167 |
| 1971 | 926 | 85 | 35 | 239 | 20 | 195 | 143 | 40 | 169 |
| 1972 | 958 | 86 | 37 | 246 | 19 | 200 | 156 | 44 | 171 |
| 1973 | 1 019 | 93 | 41 | 268 | 17 | 211 | 167 | 47 | 173 |
| 1974 | 991 | 103 | 43 | 247 | 14 | 208 | 155 | 44 | 176 |
| 1975 | 986 | 93 | 41 | 248 | 12 | 211 | 157 | 47 | 178 |
| 1976 | 1 028 | 99 | 44 | 265 | 10 | 231 | 159 | 38 | 181 |
| 1977 | 1 023 | 99 | 46 | 261 | 6 | 225 | 166 | 37 | 183 |
| 1978 | 1 060 | 102 | 44 | 277 | 5 | 229 | 177 | 41 | 185 |
| 1979 | 1 113 | 100 | 44 | 302 | 2 | 241 | 187 | 49 | 188 |
| 1980 | 1 096 | 90 | 40 | 323 | 2 | 232 | 175 | 46 | 189 |
| 1981 | 1 060 | 78 | 34 | 306 | 0 | 241 | 166 | 44 | 192 |
| 1982 | 1 048 | 71 | 30 | 313 | 0 | 234 | 164 | 41 | 194 |
| 1983 | 1 053 | 74 | 31 | 297 | 0 | 235 | 170 | 49 | 197 |
| 1984 | 1 084 | 80 | 33 | 305 | 0 | 245 | 168 | 54 | 200 |
| 1985 | 1 121 | 81 | 31 | 329 | 0 | 257 | 171 | 49 | 203 |
| 1986 | 1 122 | 73 | 29 | 329 | 0 | 258 | 176 | 50 | 206 |
| 1987 | 1 160 | 73 | 30 | 337 | 0 | 278 | 180 | 54 | 209 |
| 1988 | 1 177 | 73 | 31 | 350 | 0 | 267 | 192 | 52 | 212 |
| 1989 | 1 181 | 74 | 32 | 323 | 0 | 282 | 202 | 53 | 215 |
| 1990 | 1 200 | 74 | 34 | 327 | 0 | 301 | 201 | 46 | 217 |
| 1991 | 1 269 | 76 | 35 | 355 | 0 | 316 | 223 | 45 | 220 |
| 1992 | 1 234 | 67 | 32 | 342 | 0 | 298 | 223 | 49 | 223 |
| 1993 | 1 255 | 72 | 37 | 342 | 0 | 307 | 226 | 45 | 226 |
| 1994 | 1 246 | 73 | 34 | 318 | 0 | 316 | 227 | 47 | 229 |
| 1995 | 1 310 | 77 | 36 | 348 | 0 | 341 | 233 | 43 | 232 |
| 1996 | 1 376 | 78 | 37 | 379 | 0 | 338 | 257 | 50 | 235 |
| 1997 | 1 374 | 87 | 38 | 359 | 0 | 348 | 245 | 59 | 239 |
| 1998 | 1 396 | 86 | 38 | 359 | 0 | 341 | 275 | 55 | 242 |
| 1999 | 1 416 | 79 | 35 | 367 | 0 | 365 | 269 | 55 | 245 |
| 2000 | 1 414 | 84 | 35 | 361 | 0 | 350 | 284 | 54 | 247 |
| 2001 | 1 459 | 87 | 31 | 383 | 0 | 352 | 300 | 58 | 248 |
| 2002 | 1 446 | 73 | 27 | 348 | 0 | 369 | 323 | 56 | 250 |
| 2003 | 1 452 | 51 | 29 | 386 | 0 | 345 | 360 | 30 | 251 |
| 2004 | 1 488 | 54 | 30 | 378 | 0 | 371 | 373 | 31 | 253 |
| 2005 | 1 535 | 60 | 32 | 393 | 0 | 380 | 384 | 31 | 254 |
| 2006 | 1 499 | 56 | 31 | 384 | 0 | 370 | 368 | 35 | 256 |
| 2007 | 1 473 | 54 | 31 | 364 | 0 | 369 | 372 | 27 | 257 |
| 2008 | 1 485 | 57 | 31 | 381 | 0 | 374 | 357 | 27 | 258 |
| 2009 | 1 451 | 51 | 29 | 372 | 0 | 376 | 341 | 24 | 259 |
| 2010 | 1 522 | 54 | 30 | 409 | 0 | 395 | 350 | 24 | 259 |
| 2011 | 1 468 | 59 | 30 | 381 | 0 | 371 | 344 | 23 | 260 |
| 2012 | 1 495 | 59 | 29 | 380 | 0 | 401 | 340 | 24 | 261 |

| Table S3: useful work supply by type, 1900-2002 [units: PJ] | | | | | | | | | |
|---|---|---|---|---|---|---|---|---|---|
| **year** | **total** | **high temp. heat** | **medium temp. heat** | **low temp. heat** | **light** | **electricity** | **other prime movers** | **non-fuel** | **muscle work** |
| 1900 | 20 | 1 | 1 | 6 | 0 | 1 | 2 | 0 | 8 |
| 1901 | 20 | 2 | 1 | 6 | 0 | 1 | 2 | 0 | 8 |
| 1902 | 21 | 2 | 1 | 6 | 0 | 1 | 3 | 0 | 8 |
| 1903 | 21 | 2 | 1 | 7 | 0 | 1 | 3 | 0 | 8 |
| 1904 | 22 | 2 | 1 | 7 | 0 | 1 | 3 | 0 | 8 |
| 1905 | 22 | 2 | 1 | 7 | 0 | 1 | 3 | 0 | 8 |
| 1906 | 23 | 2 | 1 | 7 | 0 | 1 | 3 | 0 | 8 |
| 1907 | 23 | 2 | 1 | 7 | 0 | 2 | 3 | 0 | 8 |
| 1908 | 24 | 2 | 1 | 7 | 0 | 2 | 3 | 0 | 8 |
| 1909 | 24 | 2 | 1 | 8 | 0 | 2 | 3 | 0 | 8 |
| 1910 | | | | | | | | | |
| 1911 | | | | | | | | | |
| 1912 | | | | | | | | | |
| 1913 | | | | | | | | | |
| 1914 | | | | | | | | | |
| 1915 | | | | | | | | | |
| 1916 | | | | | | | | | |
| 1917 | | | | | | | | | |
| 1918 | | | | | | | | | |
| 1919 | 11 | 0 | 0 | 1 | 0 | 2 | 0 | 0 | 8 |
| 1920 | 17 | 1 | 1 | 3 | 0 | 3 | 2 | 0 | 7 |
| 1921 | 19 | 2 | 1 | 4 | 0 | 3 | 2 | 0 | 7 |
| 1922 | 20 | 2 | 1 | 5 | 0 | 3 | 2 | 0 | 7 |
| 1923 | 19 | 2 | 1 | 4 | 0 | 3 | 2 | 0 | 7 |
| 1924 | 21 | 2 | 1 | 5 | 0 | 3 | 3 | 0 | 7 |
| 1925 | 21 | 2 | 1 | 5 | 0 | 3 | 2 | 0 | 7 |
| 1926 | 21 | 2 | 1 | 5 | 0 | 4 | 2 | 0 | 7 |
| 1927 | 22 | 2 | 1 | 5 | 0 | 4 | 3 | 0 | 7 |
| 1928 | 23 | 2 | 1 | 6 | 0 | 4 | 3 | 0 | 7 |
| 1929 | 25 | 3 | 2 | 7 | 0 | 4 | 3 | 0 | 7 |
| 1930 | 22 | 2 | 1 | 5 | 0 | 4 | 2 | 0 | 7 |
| 1931 | 21 | 2 | 1 | 5 | 0 | 4 | 2 | 0 | 7 |
| 1932 | 19 | 1 | 1 | 5 | 0 | 4 | 2 | 0 | 7 |
| 1933 | 19 | 1 | 1 | 4 | 0 | 4 | 2 | 0 | 6 |
| 1934 | 19 | 2 | 1 | 4 | 0 | 4 | 2 | 0 | 6 |
| 1935 | 20 | 2 | 1 | 4 | 0 | 4 | 2 | 0 | 6 |
| 1936 | 19 | 2 | 1 | 4 | 0 | 5 | 2 | 0 | 6 |
| 1937 | 20 | 2 | 1 | 4 | 0 | 5 | 2 | 0 | 6 |
| 1938 | 21 | 2 | 1 | 4 | 0 | 5 | 2 | 0 | 6 |
| 1939 | | | | | | | | | |
| 1940 | | | | | | | | | |
| 1941 | | | | | | | | | |
| 1942 | | | | | | | | | |
| 1943 | | | | | | | | | |
| 1944 | | | | | | | | | |
| 1945 | | | | | | | | | |
| 1946 | 18 | 2 | 1 | 2 | 0 | 6 | 1 | 0 | 6 |
| 1947 | 20 | 2 | 1 | 3 | 0 | 7 | 2 | 0 | 6 |
| 1948 | 28 | 4 | 2 | 4 | 0 | 9 | 2 | 0 | 6 |
| 1949 | 30 | 5 | 3 | 5 | 0 | 9 | 2 | 0 | 6 |
| 1950 | 32 | 6 | 3 | 5 | 0 | 10 | 3 | 0 | 6 |
| 1951 | 38 | 7 | 4 | 6 | 0 | 11 | 4 | 0 | 6 |
| 1952 | 38 | 7 | 3 | 5 | 0 | 12 | 5 | 0 | 6 |
| 1953 | 40 | 7 | 4 | 6 | 0 | 12 | 6 | 0 | 6 |
| 1954 | 44 | 8 | 4 | 6 | 0 | 13 | 7 | 0 | 6 |
| 1955 | 49 | 9 | 4 | 7 | 0 | 15 | 8 | 0 | 5 |
| 1956 | 52 | 9 | 5 | 7 | 0 | 16 | 9 | 0 | 5 |
| 1957 | 54 | 10 | 5 | 7 | 0 | 17 | 9 | 0 | 5 |
| 1958 | 55 | 10 | 5 | 7 | 0 | 19 | 9 | 0 | 5 |
| 1959 | 59 | 11 | 5 | 7 | 0 | 20 | 11 | 0 | 4 |

| Table S3 (continued): useful work supply by type, 1900-2002 [units: PJ] | | | | | | | | | |
|---|---|---|---|---|---|---|---|---|---|
| year | total | high temp. heat | medium temp. heat | low temp. heat | light | electricity | other prime movers | non-fuel | muscle work |
| 1960 | 68 | 12 | 3 | 9 | 0 | 21 | 12 | 6 | 4 |
| 1961 | 69 | 12 | 3 | 8 | 0 | 22 | 13 | 7 | 4 |
| 1962 | 75 | 13 | 4 | 9 | 0 | 23 | 15 | 9 | 4 |
| 1963 | 80 | 13 | 4 | 10 | 0 | 23 | 16 | 10 | 3 |
| 1964 | 86 | 15 | 4 | 11 | 0 | 25 | 17 | 11 | 3 |
| 1965 | 92 | 15 | 4 | 11 | 0 | 29 | 18 | 11 | 3 |
| 1966 | 100 | 16 | 5 | 11 | 0 | 31 | 20 | 14 | 3 |
| 1967 | 102 | 15 | 4 | 12 | 0 | 32 | 22 | 15 | 3 |
| 1968 | 108 | 16 | 5 | 13 | 0 | 33 | 23 | 15 | 3 |
| 1969 | 114 | 18 | 5 | 14 | 0 | 31 | 26 | 18 | 3 |
| 1970 | 126 | 17 | 4 | 16 | 0 | 39 | 27 | 20 | 3 |
| 1971 | 125 | 18 | 5 | 16 | 0 | 35 | 29 | 20 | 2 |
| 1972 | 133 | 18 | 6 | 17 | 0 | 36 | 32 | 22 | 2 |
| 1973 | 143 | 20 | 6 | 18 | 0 | 39 | 35 | 23 | 2 |
| 1974 | 145 | 22 | 7 | 17 | 0 | 43 | 32 | 22 | 2 |
| 1975 | 147 | 20 | 6 | 17 | 0 | 45 | 33 | 23 | 2 |
| 1976 | 145 | 22 | 7 | 18 | 0 | 43 | 34 | 19 | 2 |
| 1977 | 151 | 22 | 7 | 18 | 0 | 49 | 35 | 19 | 2 |
| 1978 | 158 | 23 | 7 | 18 | 0 | 49 | 38 | 21 | 2 |
| 1979 | 170 | 23 | 7 | 19 | 0 | 55 | 40 | 24 | 2 |
| 1980 | 163 | 20 | 7 | 19 | 0 | 55 | 37 | 23 | 2 |
| 1981 | 159 | 18 | 6 | 17 | 0 | 58 | 35 | 22 | 2 |
| 1982 | 154 | 16 | 5 | 17 | 0 | 58 | 35 | 21 | 2 |
| 1983 | 159 | 17 | 6 | 16 | 0 | 58 | 36 | 25 | 2 |
| 1984 | 163 | 19 | 6 | 16 | 0 | 57 | 36 | 27 | 2 |
| 1985 | 167 | 19 | 6 | 17 | 0 | 62 | 37 | 25 | 2 |
| 1986 | 167 | 18 | 6 | 16 | 0 | 62 | 38 | 25 | 2 |
| 1987 | 178 | 17 | 6 | 17 | 0 | 70 | 39 | 27 | 2 |
| 1988 | 179 | 18 | 6 | 16 | 0 | 70 | 42 | 26 | 2 |
| 1989 | 183 | 18 | 6 | 15 | 0 | 70 | 45 | 27 | 2 |
| 1990 | 177 | 18 | 7 | 16 | 0 | 66 | 45 | 23 | 2 |
| 1991 | 185 | 19 | 7 | 17 | 0 | 67 | 50 | 22 | 2 |
| 1992 | 188 | 17 | 7 | 16 | 0 | 71 | 50 | 24 | 2 |
| 1993 | 192 | 18 | 8 | 16 | 0 | 75 | 51 | 22 | 2 |
| 1994 | 192 | 19 | 7 | 15 | 0 | 74 | 52 | 23 | 2 |
| 1995 | 199 | 20 | 8 | 16 | 0 | 78 | 53 | 22 | 2 |
| 1996 | 206 | 20 | 8 | 17 | 0 | 74 | 60 | 25 | 2 |
| 1997 | 214 | 23 | 8 | 17 | 0 | 77 | 57 | 30 | 2 |
| 1998 | 220 | 22 | 8 | 17 | 0 | 78 | 65 | 28 | 2 |
| 1999 | 222 | 21 | 8 | 16 | 0 | 84 | 63 | 27 | 2 |
| 2000 | 228 | 22 | 8 | 16 | 0 | 85 | 67 | 27 | 2 |
| 2001 | 232 | 23 | 7 | 17 | 0 | 83 | 71 | 29 | 2 |
| 2002 | 231 | 20 | 6 | 15 | 0 | 84 | 76 | 28 | 2 |
| 2003 | 217 | 14 | 6 | 17 | 0 | 77 | 85 | 15 | 2 |
| 2004 | 228 | 14 | 7 | 16 | 0 | 84 | 88 | 15 | 2 |
| 2005 | 235 | 16 | 7 | 17 | 0 | 86 | 91 | 15 | 2 |
| 2006 | 231 | 15 | 7 | 16 | 0 | 86 | 88 | 18 | 2 |
| 2007 | 228 | 15 | 7 | 15 | 0 | 87 | 89 | 13 | 2 |
| 2008 | 228 | 15 | 7 | 15 | 0 | 89 | 86 | 14 | 2 |
| 2009 | 221 | 14 | 6 | 15 | 0 | 91 | 81 | 12 | 2 |
| 2010 | 229 | 15 | 7 | 16 | 0 | 93 | 84 | 12 | 2 |
| 2011 | 221 | 16 | 7 | 15 | 0 | 88 | 82 | 11 | 2 |
| 2012 | 231 | 16 | 7 | 14 | 0 | 99 | 81 | 12 | 2 |

## Data Sources

### *IEA database, 1960-2012*

The International Energy Agency (IEA, United Nations, 2013) provides a database where all relevant energy flows of all countries in the world are covered for the years 1960 to 2002. We used data for all energy carriers (primary and secondary products of coal, oil, gas, renewables) and their uses along specific flow categories. The quantities in the IEA database are reported in ktoe (kiloton of oil equivalent). To convert ktoe to TJ we used the coefficient defined in the Energy Statistics Manual (OECD/IEA, 2005): 41,87 TJ/ktoe.

Since the IEA database provides the final use in industrial sectors most consistently for all energy carriers, we used the IEA data for the year 1960 onwards. Other data sources were used for the years prior to 1960.

### *Historical data sources, 1900-1960*

For the historical data we had several data sources of different quality at hand:

- Statistische Handbücher (Bundesamt für Statistik, 1925 and other years)
- Wirtschafts- und sozialstatistisches Handbuch (KAAW, 1970)
- Statistische Reihen zur österreichischen Wirtschaftsgeschichte (Butschek et al., 1998)
- Energiebericht 1990 (BMWA, 1990)

– Krausmann et al. (2003): Vergleichende Untersuchung zur langfristigen Entwicklung von gesellschaftlichem Stoffwechsel und Landnutzung in Österreich und dem Vereinigten Königreich.

In the following we will give a short presentation of the sources and the data contained:

***Source 1: Statistische Handbücher*** (Bundesamt für Statistik, 1925 and other years)

The statistical handbook is a yearly publication of the Austrian Statistical Office. These yearbooks include data on energy production, imports and exports, as well as supplies to 6 demand categories: (1) transport, (2) gas and hydro-power plants, (3) electricity plants, (4) households, (5) industry, (6) use of occupying forces. The industry sector is further divided into 18 sectors. Data are available for the years 1922, 1924-1937, and 1947-1958. The energy carriers included are: hard coal and brown coal, coking coal, crude oil and derived products (in tonnes); natural gas and town gas (in cubic metres).

***Source 2: Wirtschafts- und sozialstatistisches Handbuch 1945-1969*** (KAAW, 1970)

The "Wirtschafts- und sozialstatistisches Handbuch" is a publication of the Austrian Chamber of Workers and Employees and covers extraction, production, and trade, as well as uses along 6 categories: (1) transport, (2) electricity production, (3) production of gas, (4) mining and industry, (5) uses in coking plants, (6) households. The time period covered is 1925, 1929, 1930, 1935, 1937, 1945-1969. The energy carriers included are: hard coal and brown coal, coking coal reported (in hard coal

equivalents); crude oil and derived products (in tonnes); and natural gas (in cubic metres; consumption in hard coal equivalents).

***Source 3: Statistische Reihen zur österreichischen Wirtschaftsgeschichte*** (Butschek et al., 1998)

This publication of the WIFO, the Austrian Institute of Economic Research, covers production and total consumption for the time period 1921-1996. The energy carriers included are: hard coal, brown coal, other coal, and crude oil in TJ.

***Source 4: Energiebericht 1990*** (BMWA, 1990)

The "Energiebericht 1990" (energy report) was published by the Austrian Ministry of Economics and Labour and contains energy data on production, imports, exports and total consumption for the years 1955-1988. The energy carriers included are: coal (total), oil (and derived products for exports) in TJ, and natural gas and town gas in cubic metres.

***Source 5: Krausmann et al. (2003)***

In this publication "Vergleichende Untersuchung zur langfristigen Entwicklung von gesellschaftlichem Stoffwechsel und Landnutzung in Österreich und dem Vereinigten Königreich" the authors issued a historical time series (1000-1995) for the Austrian production, imports and exports of hard coal, lignite, oil in tonnes, and production for gas in cubic metres. The data are partly based on previous mentioned data sources but for the early years of the 20$^{th}$ century, estimations were applied.

### *Conversion factors*

For the conversion of ktoe (kiloton of oil equivalent) to TJ we used a coefficient defined in the IEA handbook (OECD/IEA, 2005): 41,87 TJ/ktoe.

For the conversion of other units to TJ we used the calorific values of Statistics Austria (Bittermann, 2004) of the earliest year available, i.e. 1970.

### *Comparison of data sources*

A comparison of the historical data sources showed good congruence both between the different historical data sources but also with data from the IEA; see Figure S1. Major problems were only given for the years around the First and Second World War. In these years, data are only partly available.

**Figure S1: Comparison of historical data sources – selected examples**

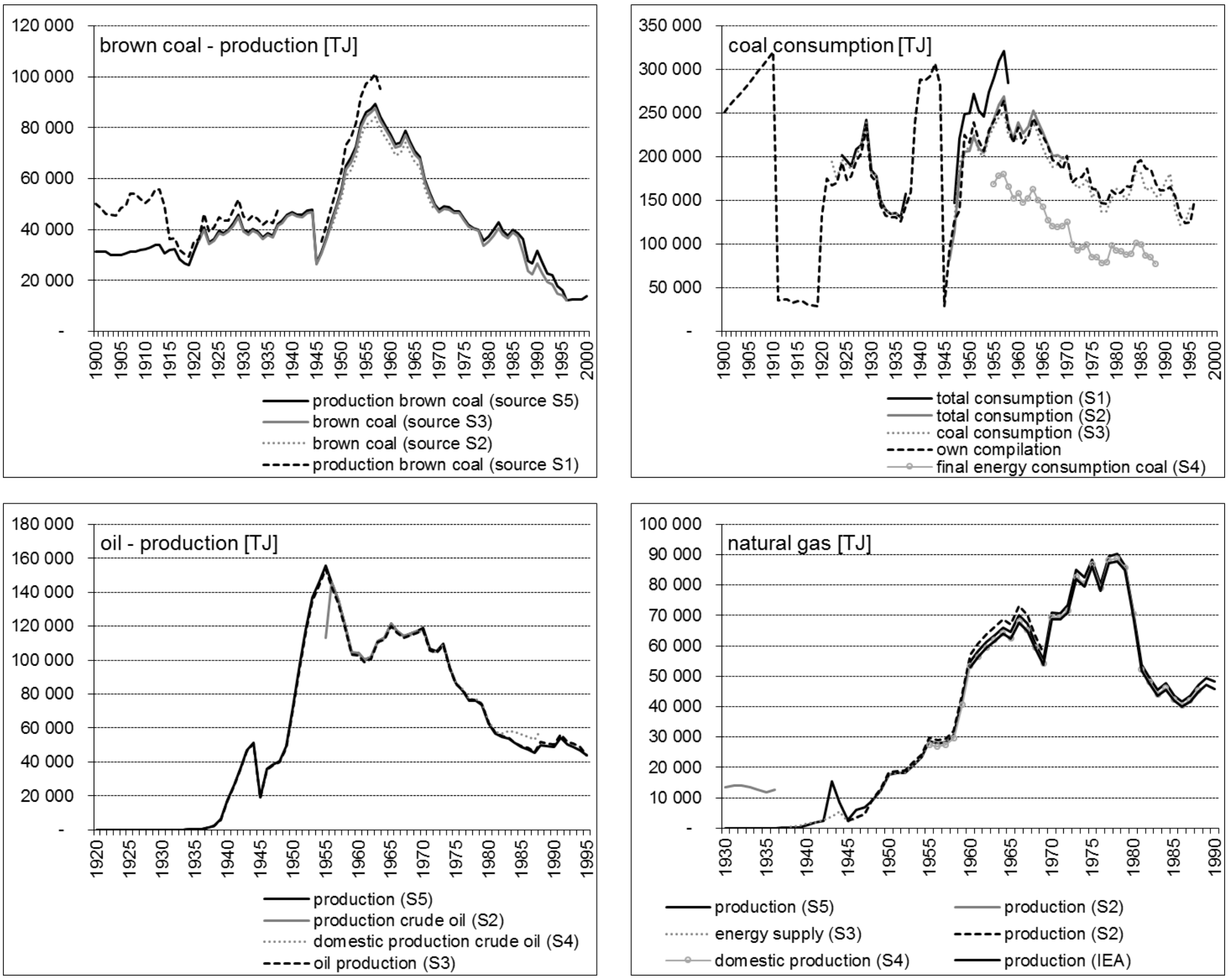

### *Final data set*

Data for the years 1960 to 2002 were taken from the IEA database. For the previous years the final data set was a compilation of the following sources:

**Table S4: final dataset, 1900-1960**

| | | |
|---|---|---|
| **Hard coal: production** | **1900-1938** | **Statistische Handbücher (Bundesamt für Statistik, 1925 and other years)** |
| | **1938-1960** | **WIFO (Butschek et al., 1998)** |
| **Brown coal: production** | **1900-1920** | **Krausmann et al. (2003)** |
| | **1920-1960** | **WIFO (Butschek et al., 1998)** |
| **Coal: imports, export** | | **Krausmann et al. (2003)** |
| **Coal: consumption** | **1900-1920** | **Calculated as production + imports – exports** |
| | **1920-1960** | **WIFO (Butschek et al., 1998)** |
| **Oil: production** | | **WIFO (Butschek et al., 1998)** |
| **Oil: imports** | **1951-1954** | **Krausmann et al. (2003)** |
| | **1955-1960** | **BMWA (1990)** |
| **Oil: exports** | **1951-1954** | **Trend: Krausmann et al. (2003), level: BMWA (1990)** |
| | **1955-1960** | **BMWA (1990)** |
| **Oil: consumption** | **1932-1954** | **Trend: Krausmann et al. (2003), level: BMWA (1990)** |
| | **1955-1960** | **BMWA (1990)** |
| **Gas: production** | | **WIFO (Butschek et al., 1998)** |
| **Gas: imports and exports** | | **None before 1960** |
| **Gas: consumption** | | **WIFO (Butschek et al., 1998)** |

### *Sectoral breakdown for the historical time series*

The sectoral breakdown for coal consumption was taken from the statistical handbooks. For missing information previous 1924, we assumed the same allocation among sectors as in 1924. For the years 1937 to 1947 we decided to apply the distribution among sectors as the calculated mean of the years 1937 and 1947.

In the case of oil the statistical source provided no information on consumption along different use categories. In this case and due to the fact that oil consumption only gained importance from the 1950s onwards, we applied the same shares as in the year 1960, which is the first year of the IEA time series. The same procedure was applied to natural gas.

## References

Bittermann, W., 2004. Energiebilanzen Österreich 1970-2004. Statistik Austria, Wien.

BMWA, 1990. Energiebericht 1990 der österreichischen Bundesregierung. Bundesministerium für wirtschaftliche Angelegenheiten, Wien.

Bundesamt für Statistik, 1925. Statistisches Handbuch für die Republik Österreich. Bundesamt für Statistik, Wien.

Butschek, F., Predl, M., Steiner, C., 1998. Statistische Reihen zur österreichischen Wirtschaftsgeschichte, Die österreichische Wirtschaft seit der industriellen Revolution. Österreichisches Institut für Wirtschaftsforschung (WIFO), Wien.

KAAW, 1970. Wirtschafts- und sozialstatistisches Handbuch 1945-1969. Kammer für Arbeiter und Angestellte für Wien, Wien.

Krausmann, F., Schandl, H., Schulz, N.B., 2003. Vergleichende Untersuchung zur langfristigen Entwicklung von gesellschaftlichem Stoffwechsel und Landnutzung in Österreich und dem Vereinigten Königreich. Breuninger Stiftung, Stuttgart.

OECD/IEA, 2005. Energy Statistics Manual. International Energy Agency (IEA), Paris.

United Nations, 2013. IEA - International Energy Agency - statistics [WWW Document]. URL http://www.iea.org/statistics/ (accessed 11.12.14).
